# Early computed tomography-based scores to predict decompressive hemicraniectomy after endovascular therapy in acute ischemic stroke

**DOI:** 10.1371/journal.pone.0173737

**Published:** 2017-03-10

**Authors:** Ilko L. Maier, Daniel Behme, Marlena Schnieder, Ioannis Tsogkas, Katharina Schregel, Mathias Bähr, Michael Knauth, Jan Liman, Marios-Nikos Psychogios

**Affiliations:** 1 Department of Neurology, University Medical Center Goettingen, Goettingen, Germany; 2 Department of Neuroradiology, University Medical Center Goettingen, Goettingen, Germany; University of Münster, GERMANY

## Abstract

**Background:**

Identification of patients requiring decompressive hemicraniectomy (DH) after endovascular therapy (EVT) is crucial as clinical signs are not reliable and early DH has been shown to improve clinical outcome. The aim of our study was to identify imaging-based scores to predict the risk for space occupying ischemic stroke and DH.

**Methods:**

Prospectively derived data from patients with acute large artery occlusion within the anterior circulation and EVT was analyzed in this monocentric study. Predictive value of non-contrast cranial computed tomography (ncCT) and cerebral blood volume (CBV) Alberta Stroke Program Early CT score (ASPECTS) were investigated for DH using logistic regression models and Receiver Operating Characteristic Curve analysis.

**Results:**

From 218 patients with EVT, DH was performed in 20 patients (9.2%). Baseline- (7 vs. 9; p = 0.009) and follow-up ncCT ASPECTS (1 vs. 7, p<0.001) as well as baseline CBV ASPECTS (5 vs. 7, p<0.001) were significantly lower in patients with DH. ncCT (baseline: OR 0.71, p = 0.018; follow-up: OR 0.32, p = <0.001) and CBV ASPECTS (OR 0.63, p = 0.008) predicted DH. Cut-off ncCT-ASPECTS on baseline was 7-, ncCT-ASPECTS on follow-up was 4- and CBV ASPECTS on baseline was 5 points.

**Conclusions:**

ASPECTS could be useful to early identify patients requiring DH after EVT for acute large vessel occlusion.

## Introduction

Endovascular therapy (EVT) with stent-retriever devices in acute ischemic stroke involving the anterior circulation has been shown to be superior compared to standard medical treatment in recent randomized trials [1–5]. A meta-analysis of these trials showed benefits of endovascular therapy in almost all patient subgroups, while overall recanalization rates of 71% have been reported [6]. These studies suggest a decrease in rates of decompressive hemicraniectomy (DH) in the future, which has already been reported in a retrospective study by Sporns et al, who found a significant reduction in rates of DH after introduction of EVT between 2009 and 2013 in 497 patients with proximal arterial occlusion (17.4 vs 8.2%) [7].

DH has been shown to improve clinical outcome, shortens in-patient stay and mortality of patients with space occupying ischemic stroke [8, 9]. There is evidence that DH should be performed early and clinicians should not wait for clinical deterioration (e.g. decrease in consciousness) or radiological signs (e.g. midline-shift) [8, 10]. Patients at risk should be identified reliably and as early as possible, because there are neither validated clinical signs nor every patient can be extubated promptly and judged adequately after EVT (e.g. due to aspiration, pulmonary co-morbidities or postinterventional delirium).

The recent meta-analysis of the five thrombectomy trials showed that even patients with lower baseline Alberta Stroke Program Early CT score (ASPECTS), which quantifies infarct demarcation, can benefit from EVT [6]. However, not only patients with unsuccessful EVT, but also patients with low ASPECTS at baseline and follow-up are at risk of developing space-occupying infarctions. The extent of pretreatment infarction at baseline is a predictor for clinical outcome in patients with EVT [11, 12]. In addition, poor collateralization might also increase the risk for space occupying stroke. Therefore, we investigated the predictive value of non-contrast cranial computed tomography (ncCT) ASPECTS, cerebral blood volume (CBV) ASPECTS and baseline Menon score, a collateral score which can be used to determine extent of cerebral collateralization, for DH after EVT.

## Materials and methods

### Patient population

Clinical and neuroradiological data were analyzed from a prospectively derived, monocentric database including neuroradiological and neurological information of interventional treatment and clinical outcome. Ethics approval was sought from the ethics committee of the University Medical Center Goettingen and all patients gave informed written consent for the anonymized use of disease-related data on hospitalization. Patients were included in the analysis when presenting with acute ischemic stroke of the anterior circulation and receiving EVT between January 2013 and November 2016. Periprocedural factors were recorded by a stroke-experienced senior neuroradiologist and clinical data has been evaluated by an experienced, stroke-trained neurologist.

### Imaging based scores

ASPECTS were separately assessed by two neuroradiologists (one with more than 5 years of experience). If ASPECTS differed between the raters, the neuroradiologists reviewed the imaging together and sought consensus. They separately rated ncCT and CT-perfusion (CTP) scans with the ASPECTS, a 10-point scoring system of the middle cerebral artery (MCA) territory. For every MCA region with acute ischemic signs, 1 point is subtracted from 10, resulting in an ASPECTS of 10 for a scan without ischemic lesions and an ASPECTS of 0 for complete MCA infarction [13]. The Menon collateral score (CS) quantifies pial collateral filling on single phase CT angiography comparing the symptomatic- and asymptomatic hemisphere. It ranges from 0 (no vessels visible within the ischemic territory) to 5 (increased or normal prominence and extent of pial vessels within the ischemic territory). In our study, we used this score in its recent description, which quantifies the pial arterial filling from the anterior and posterior circulation, respectively, and adds the values to a 0 (no collaterals) to 10 (increased or normal collaterals) ordinal score [14].

### Multidisciplinary stroke treatment

EVT was performed if patients presented within 6 hours after symptom onset, large vessel occlusion was found in CT-angiography (CTA), significant cerebral blood flow (CBF)/CBV-mismatch on multimodal stroke imaging (defined as difference of ≥ 2 points between CBF ASPECTS and CBV ASPECTS) and intracranial hemorrhage had been ruled out. Decision to perform EVT was based on a standard operating procedure [15] after January 2015 while being based on the judgement of the treating senior neurologist and neuroradiologist bevor January 2015. I.v.-recombinant tissue plasminogen activator was administered right after the ncCT if contraindications had been ruled out (0.9 mg/kg over 1 hour with 10% of initial bolus) and EVT has been performed without delay (bridging therapy). Following stroke imaging, patients were transferred to the angiography suite (Artis Zee (until 2015) and Artis Q (since 2015), Siemens Healthcare, Forchheim, Germany) where they underwent mechanical thrombectomy either with an aspiration catheter or with a combination of aspiration catheters and retrievable stents (Aperio, Acandis, Pforzheim, Germany; Trevo, Stryker, Mountain View, California; 3D Separator, Penumbra).

In the present study, baseline ASPECTS on ncCT was determined in all patients. ASPECTS on whole-brain CTP and CS could be evaluated in a subgroup (due to missing data, technical or procedural problems). These patients presented with occlusion of the proximal-, distal internal carotid- or medial cerebral artery (MCA) in the M1 or M2-segment, which was diagnosed on initial CT-angiogram. Vessel occlusion was confirmed on subsequent digital subtraction angiography and defined as modified thrombolysis in cerebral infarction score (mTICI) of 0 or 1. Successful recanalization was defined as mTICI 2b-3 on the final angiogram [16]. Patients were excluded if baseline imaging showed ICH or if DH had to be performed because of symptomatic ICH instead of space occupying cerebral infarction.

Indication for DH was based on an interdisciplinary case-to-case decision including the following factors: (suspected) patient will, co-morbidity, age, ischemic stroke within 48 h before admission, (anticipated) infarct-demarcation in more than two-thirds of the MCA territory, ncCT ASPECTS ≤ 4, space occupying edematous infarct-swelling and clinical deterioration. All patients with DH received a follow-up ncCT before surgery either as regular control ncCT 24 hours after EVT or earlier if clinical deterioration had been noted.

### Imaging

Stroke imaging was acquired with a 128-slice multidetector CT scanner (Siemens Definition AS+; Siemens Healthcare Sector, Forchheim, Germany) and included a ncCT, followed by near whole-brain CTP and single phase CTA of extra- and intracranial arteries. The scan-protocol has been described in detail elsewhere [17].

### Statistical analysis

Statistical analysis was performed using SPSS 21 (IBM SPSS Statistics, Armonk, NY, USA). Characteristics of all patients are shown as mean ± standard deviation (SD) if normally distributed and as median interquartile range (IQR) if not. Comparisons of imaging-based stroke scores between patients with- and without DH were performed using the Mann-Whitney-U-test and Fischer's exact-test as appropriate. To assess the predictive value of imaging-based stroke scores, logistic regression models including success of recanalization, symptom-to-final angiogram time, age and National Institute of Health Stroke Scale (NIHSS) have been used. Area under the receiver operator curve (AUROC) and confidence intervals (CI) were calculated for every imaging-based stroke score for the endpoint DH. AUROC vary from 0.5 for a model that correctly predicts outcome no better than chance to 1.0 for a model that perfectly discriminates between endpoints. Cut-off scores were defined as scores with maximal Youden-Index. P-values below 0.05 were considered statistically significant.

## Results

**Fig 1:**
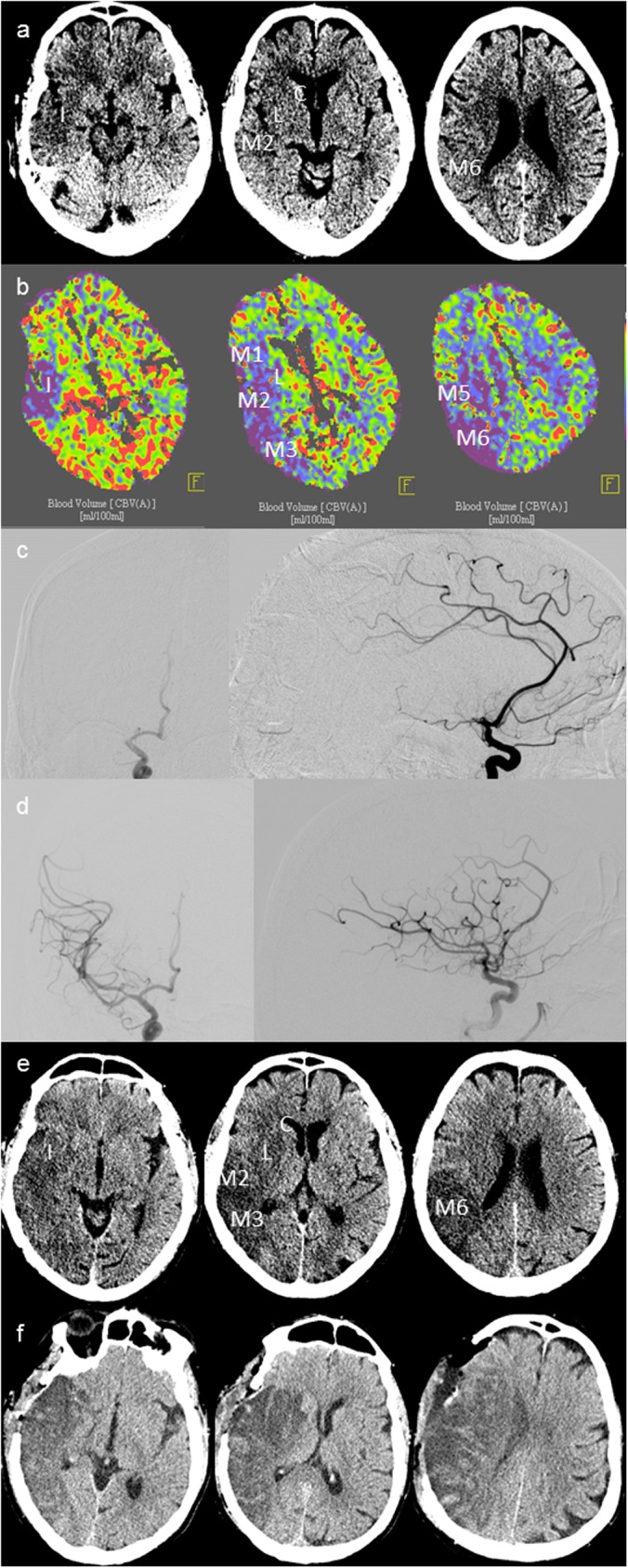
Multimodal stroke imaging from a patient with endovascular therapy and decompressive hemicraniectomy CT-slices are shown in insular (left), ganglionic (middle) and supraganglionic levels from left to right. (a) ncCT on baseline presentation with an ASPECTS of 5, a symptom-to-CT time of 118 min and a baseline NIHSS of 12. (b) Cerebral blood volume on baseline with an ASPECTS of 3. (c) DSA before EVT with mTICI 0 and (d) after EVT with successful recanalization. (e) follow up ncCT with temporal edematous swelling compressing the right anterior horn of the lateral ventricle. (f) ncCT after DH with infarct demarcation of the territorial infarction of the medial cerebral artery circulation. ncCT: non-contrast cranial computed tomography; ASPECTS: Alberta Stroke Program Early CT score; NIHSS: National Institute of Health Stroke Scale; DSA: digital subtraction angiography; EVT: endovascular therapy; mTICI: modified thrombolysis in cerebral infarction score.

From 218 included patients with EVT for large vessel occlusion in the anterior circulation, DH was carried out in 20 (9.2%) patients within a mean period from baseline imaging to surgery of 40.5 ± 37.4 hours (mean ± standard deviation; Table 1). Patients in the DH-group were significantly younger (63 vs 76 years; p = <0.001), had a lower rate of hyperlipoproteinemia (20% vs 50%; p = 0.029) and tended to have a higher NIHSS (18 vs 16 points; p = 0.095). Concerning periprocedural factors of EVT, patients in the DH-group had a lower rate of successful recanalization (40% vs 72%; p = 0.005) and a longer symptom-to-final angiogram time (270 vs 228 min; p = 0.036). A representative case of a patient with successful racemization requiring DH is presented in Fig 1.

**Table 1:** Baseline characteristics of patients with- and without hemicraniectomy

			
	Hemicraniectomy (n = 20)	No-hemicraniectomy (n = 198)	p-value*
Age (years, IQR)	63 (48–67.5)	75.5 (64–80)	<0.001
Sex (male, %)	9 (45)	98 (49.5)	0.816
Hypertension (n, %)	14 (70)	162 (81.8)	0.233
HLP (n, %)	4 (20)	98 (49.5)	0.029
Diabetes mellitus (n, %)	6 (30)	52 (26.3)	0.596
Atrial fibrillation (n, %)	6 (30)	87 (43.9)	0.339
IV-thrombolysis (n, %)	11 (55)	141 (71.2)	0.131
Successful recanalization (n, %)	8 (40)	143 (72.2)	0.005
NIHSS baseline (points ± SD)	18 ± 8	16 ± 6	0.095
Symptom to admission time (min, IQR)	129 (45, 210)	80 (57.25, 155)	0.480
Groin puncture to final angiogram time (min, IQR)	64 (46.75, 90.25)	53 (36, 76.5)	0.086
Symptom to final angiogram time (min, IQR)	270 (210–362)	228 (175–283.5)	0.036

IQR: Interquartile range, HLP: hyperlipoproteinemia, IV: intravenous, NIHSS: National Institute of Health Stroke Scale, SD: Standard deviation

*Mann-Whitney-U-test and Fischer's exact test as appropriate

As shown in Table 2, baseline- (7 vs 9; p = 0.009), follow-up ncCT-ASPECTS (1 vs 7, p<0.001) as well as CBV ASPECTS (5 vs 7, p<0.001) were significantly lower in patients with DH. There was also a trend towards a lower CS (4.6 vs 6 points; p = 0.054) for patients with DH. Time to follow-up ncCT was not significantly different between groups (29.5 vs 26 hours; p = 0.186).

**Table 2:** ncCT-, CBV-ASPECTS and collateral score in patients with- and without hemicraniectomy

	Hemicraniectomy (n = 20)	No-hemicraniectomy (n = 198)	p-value*
ncCT-ASPECTS at baseline (median, IQR)	7 (5–8.75)	9 (7–9)	0.009
ncCT-ASPECTS follow-up (n = 173; median, IQR)	1 (0–3)	7 (5–8)	<0.001
Time of follow-up CT (hours, IQR)	29.5 (26.5–53)	26 (18–51)	0.186
CBV-ASPECTS (n = 171; median, IQR)	5 (3.25–7)	7 (6–8)	<0.001
Collateral score (n = 116; mean ± SD)	4.56 ± 2.6	6.04 ± 2.16	0.054

IQR: Interquartile range, SD: standard deviation, ncCT: non-contrast computed tomography, CBV: cerebral blood volume, ASPECTS: Alberta Stroke Program Early CT score;

*Mann-Whitney-U-test or T-Test as appropriate

Based on the group-differences shown in Table 1, we created logistic regression models including one imaging-based stroke score respectively in combination with the confounding factors age, successful recanalization, time-to-final angiogram and baseline NIHSS (Table 3). Patients with high baseline ncCT- and follow-up ncCT-ASPECTS as well as CBV-ASPECTS had a decreased risk for DH. ncCT-ASPECTS after a median follow-up period of 27 hours (IQR, 18–48) was the strongest predictor for DH (OR 0.32; CI 95%, 0.17–0.0.59; p<0.001), followed by CBV- (OR 0.63; 0.45–0.89; p = 0.008) and baseline ncCT-ASPECTS (OR 0.71; 0.47–1.07; p = 0.018). This association was not significant for the CS and DH (OR 0.63; 0.45–0.89; p = 0.100).

**Table 3:** Logistic regression models including imaging-based stroke scores for the risk stratification for decompressive hemicraniectomy after acute major ischemic stroke

		Odds ratio (95%CI)	p-value
ncCT-ASPECTS baseline		0.71 (0.47–1.07)	**0.018**
	Recanalization successful	0.28 (0.08–0.95)	**0.041**
	Symptom-to-groin time	1 (0.99–1.01)	0.186
	Age	0.93 (0.89–0.97)	**0.001**
	NIHSS baseline	1.05 (0.96–1.14)	0.317
ncCT-ASPECTS follow-up		0.32 (0.17–0.0.59)	**<0.001**
	Recanalization successful	1.01 (0.16–6.42)	0.988
	Symptom-to-final angiogram time	1 (0.99–1.01)	0.999
	Age	0.87 (0.79–0.96)	**0.004**
	NIHSS baseline	0.936 (0.81–1.08)	0.371
CBV-ASPECTS baseline		0.63 (0.45–0.89)	**0.008**
	Recanalization successful	0.27 (0.06–1.22)	0.089
	Symptom-to-final angiogram time	1.01 (0.06–1.22)	0.081
	Age	0.92 (0.87–0.97)	**0.004**
	NIHSS baseline	1.03 (0.92–1.16)	0.560
Collateral score		0.63 (0.45–0.89)	0.100
	Recanalization successful	0.25 (0.05–1.35)	0.108
	Symptom-to-final angiogram time	1.01 (0.99–1.01)	0.455
	Age	0.96 (0.91–1.02)	0.961
	NIHSS baseline	1.13 (1–1.28)	**0.045**

ncCT: non-contrast computed tomography; CBV: cerebral blood volume; ASPECTS: Alberta Stroke Program Early CT score; NIHSS: National Institute of Health Stroke Scale

Table 4 shows the receiver operating characteristic analysis, which revealed that ncCT-ASPECTS at follow-up had excellent predictive value for DH (AUROC 0.95, CI 95%, 0.90–0.98) with a cut-off score of 4 points, followed by a moderate predictive value of the baseline ncCT- (AUROC 0.74, 0.68–0.80) and CBV-ASPECTS (AUROC 0.74, 0.67–0.81) with a cut-off score of 7 and 5 points respectively. Predictive value of the CS was poor (AUROC 0.68, 0.59–0,77) with a cut-off score of 5 points.

**Table 4:** Predictive value of imaging parameters for hemicraniectomy

	Hemicraniectomy	
	AUROC (95% CI)	Cut-of score (sens, spec)
ncCT-ASPECTS at baseline (median, IQR)	0.74 (0.68–0.80)	7 (66.67, 70.68)
ncCT-ASPECTS follow-up (n = 173; median, IQR)	0.95 (0,90–0,98)	4 (100, 79.22)
CBV-ASPECTS (n = 171; median, IQR)	0.74 (0.67–0.81)	5 (57.14, 81.08)
Collateral score (n = 116; mean ± SD)	0.68 (0.59–0,77)	5 (77.78, 59.05)

IQR: Interquartile range, SD: standard deviation, ncCT: non-contrast computed tomography, CBV: cranial blood volume, ASPECTS: Alberta Stroke Program Early CT score

## Discussion

In the present study, we investigated the prognostic value of imaging-based stroke scores to identify patients likely to require DH after EVT for large vessel occlusion in acute ischemic stroke. While there are no validated clinical signs indicating the need for DH after large, hemispheric stroke, internal carotid occlusion, female sex and age <60 years have been identified as risk factors [18–19]. A study by Kasner et al showed an association between fatal brain edema and a history of hypertension or heart failure, increased white blood cell count, involvement of multiple vascular territories and early hypodensities on ncCT in ≥ 50% of the medial cerebral artery territory [20], which was already shown in the placebo arm of a randomized study investigating the effect of lubeluzole in patients with acute major stroke [21]. In our study, we found evidence for the predictive value of ncCT- and CBV-ASPECTS to identify patients at risk to develop space occupying ischemic stroke, which could be used to more exactly quantify ischemic areas and to accompany the previously described risk factors. ASPECTS are determined early after the ischemic event and represent a quantification of tissue in which cytotoxic and vasogenic edema will occur. This edema usually develops within the first 2 and 4 days, but can develop more rapidly in the first 24 hours after stroke [22]. Therefore, early findings on stroke imaging like attenuation changes within the grey matter resulting in the loss of grey-white matter differentiation at the cortex, loss of distinction of basal ganglia and of the insular ribbon, which are included in the ASPECTS, can guide therapeutic decisions. It has been shown, that if these changes have been found in more than 50% of the MCA territory, this predicts malignant infarction with a sensitivity of 61% and a specificity of 94% [23, 24]. ASPECTS represents a more standardized approach to identify irreversibly damaged brain tissue compared to volumetric measurements of ischemic brain tissue on ncCT. In this respect, a study by MacCallum et al. already showed that an ASPECTS <7 predicts a malignant course of ischemic stroke with increase of neurological deficit and decrease in consciousness in a population of patients with territorial ischemic stroke in the MCA circulation [25]. The cut-off ncCT-ASPECTS on baseline imaging in our study as well as the sensitivity and specificity (66% and 70% vs 50% and 86%) are comparable between the study by MacCallum et al. and our study, indicating that the ASPECTS is likely to be a robust tool for risk stratification in patients with major stroke in the MCA territory.

However, the study by McCallum et al. does not report recanalization rates and did not include patients with EVT, while 27% of included patients received intravenous thrombolysis [25]. Successful recanalization is associated with improved outcome, however patients undergoing successful EVT with low baseline ASPECTS are at risk to develop space occupying infarctions anyway. In our study 8 (40%) from 20 patients in the DH-group were successfully recanalized, but required DH after all. Consequently, clinicians should not rely on successful recanalization alone as a predictor for good clinical outcome, but should include the ASPECTS for risk stratification. Moreover, showing even a higher predictive value of ncCT-ASPECTS on follow-up, our study stresses the need for regular follow-up ncCTs 24 hours after EVT, and maybe even earlier in patients showing an ASPECTS < 7 on baseline ncCT. The latest generation of flat detector CT allows for ASPECTS evaluation of the supratentorial brain within the angio suite [26] and patients with low ASPECTS on the flat detector CT or large contrast agent extravasation could benefit from earlier follow up ncCT, especially if the patents neurological status can’t be judged adequately (e.g. caused by sedative drugs and/or intubation).

Our study could also demonstrate that CBV-ASPECTS is useful for risk stratification concerning the requirement for DH. The lower cut-off at baseline compared to ncCT-ASPECTS (5 vs 7) could indicate that CBV-ASPECTS earlier identifies patients at risk for DH, as cut-off ncCT-ASPECTS at follow-up (with the highest predictive value) was comparable to CBV-ASPECTS at baseline (5 vs 4). Therefore, CBV indicates the core infarction which can`t be rescued by reperfusion therapies while changes in ncCT are more dynamic during the first 24 hours after stroke. However, our findings have to be interpreted with caution, as a recent study by Geuskens et al. [27], including patients from the MR-CLEAN trial [1], showed a significant proportion of patients with misclassification of ischemic core region and as different processing software packages include different thresholds of perfusion parameters, which again have been shown to produce different results [28, 29].

The CS described by Menon et al. is also likely to be useful for the prediction of DH-requirement [14]. In our study, there was a trend towards a lower CS in patients with DH and predictive value was lower compared to the ASPECTS. On the one hand, this could be explained by the lower patient number, in which the Menon score had been determined (n = 116) and thus a problem of statistical power; on the other hand, this score could be more variable concerning inter rater reliability and susceptibility to fluctuations of systemic blood pressure, as the autoregulation of cerebral arteries fails and perfusion (of the penumbra) is depends on mean arterial pressure [30].

Considering both the overall longer pre- and intra-clinical periods of multidisciplinary stroke treatment and lower CS in the DH-group, it is likely that collateralization as well as time from symptom onset to final angiogram and EVT influence the extent of cerebral infarction. As difference between symptom onset to admission was not significant different between the DH- and non-DH group, one could speculate that in-hospital delays of treatment and time of EVT influences the risk for DH more than pre-hospital delay, which is in line with a recent meta-analysis on periprocedural factors in the large randomized thrombectomy trials [31].

Strength of our study is the inclusion of prospectively derived patient data and the use of the robust outcome parameter DH in contrast to a clinical outcome construct like malignant infarction, which varies in its definition. The limitations of our study the monocentric design and low number of patients quantified with the CS.

In conclusion, our study provides additional evidence for the predictive value of ASPECTS obtained by initial, multimodal stroke imaging and underlines the importance of regular follow-up ncCT. Imaging information seems to bear important information for the clinician for risk stratification besides known clinical risk factors like age and large vessel occlusion. Other studies with a multicenter design are needed to confirm our findings and to further determine the best timepoint for follow-up imaging in patients at risk for the development of space-occupying infarction.
